# Treatment of Epidermal Pathology in a Pediatric Patient With Keratitis–Ichthyosis–Deafness (KID) Syndrome With Topical Mefenamic Acid

**DOI:** 10.1111/pde.70000

**Published:** 2025-08-15

**Authors:** Radhika Gupta, Alisa Ho, Lars Brichta, Albert C. Yan

**Affiliations:** ^1^ Perelman School of Medicine University of Pennsylvania Philadelphia Pennsylvania USA; ^2^ Department of Internal Medicine Changi General Hospital Simei Singapore; ^3^ Chemistry Rx Compounding and Specialty Pharmacy Folcroft Pennsylvania USA; ^4^ Dermatology Section Children's Hospital of Philadelphia Philadelphia Pennsylvania USA

**Keywords:** keratitis–ichthyosis–deafness syndrome, mefenamic acid, non‐steroidal

## Abstract

Keratitis–ichthyosis–deafness (KID) syndrome is a rare genetic condition typically presenting at birth with ichthyosiform erythroderma and bilateral hearing loss and later progressing to diffuse keratodermatous plaques with scaling. The condition is associated with mutations in the *GJB2* gene, which lead to aberrant activation of connexin hemichannels in keratinocytes. While no targeted treatment currently exists, a previously published in vivo study demonstrated that flufenamic acid (FFA), a nonspecific connexin inhibitor, reduces epidermal pathology in transgenic mouse models expressing the lethal *GJB2* mutation. Herein, we report the case of a 5‐year‐old boy with KID syndrome presenting with painful, persistent scalp lesions, which responded remarkably well to topical mefenamic acid, offering a potential novel therapy for managing this challenging condition.

## 1 Introduction

Keratitis–ichthyosis–deafness (KID) syndrome is a rare condition, first described by Skinner et al. [1] in 1981. Patients typically present at birth with red, leathery skin and subsequently develop non‐scaly, keratodermatous plaques on the face and extremities within the first year of life, alongside bilateral hearing loss, keratitis, and ichthyosiform erythroderma. Given the limited effectiveness of existing treatments, KID syndrome has a high incidence of childhood mortality due to infectious complications, increased cancer risk, and respiratory dysfunction [2, 3]. Documented treatments for skin findings in KID include hydrocolloid dressings for hyperkeratosis, isotretinoin and acitretin, systemic retinoids that have provided variable responses, and alitretinoin for dissecting cellulitis of the scalp [4, 5, 6, 7]. Management primarily involves regular ophthalmology evaluations for keratitis, dermatological surveillance for skin and mucosal malignancies, hearing aids for hearing loss, and vigorous treatment of infections, particularly for *S. aureus* , *E. coli* , *P. aeruginosa* , *T. rubrum* , and *C. albicans* infections [2, 3].

KID syndrome has been associated with aberrant connexin hemichannel activation due to mutations in *GJB2* and *GJB3*, which encode connexin 26 (Cx26) and connexin‐31, respectively [8]. Although most cases are inherited in an autosomal dominant fashion, some reports indicate autosomal recessive transmission [9, 10, 11]. De novo mutations in *GJB2* have also been identified in 13 unrelated patients with KID [12, 13]. In patients with these mutations, there is gain‐of‐function hemichannel activity in the affected keratinocytes. Thus, an increase in hemichannel activity suggests that hemichannel inhibition could be therapeutically beneficial [14]. Sellitto et al.'s [15] study demonstrated that flufenamic acid (FFA) effectively blocked hemichannel activity and reduced epidermal pathology in transgenic mouse models expressing the lethal Cx26 mutation. Although FFA is currently not available in the United States, mefenamic acid—an FDA‐approved oral medication for the treatment of mild‐to‐moderate pain—shares similar properties.

Our case presentation highlights the potential benefit of mefenamic acid as a novel, safe, efficacious and cost‐efficient topical therapy for skin findings of KID syndrome. This medication is hypothesized to exert its effects through inhibition of aberrant connexin activity in keratinocytes.

## 2 Case Presentation

We describe the case of a 5‐year‐old male who presented at birth with a range of congenital abnormalities, including ruddy‐colored skin, ichthyosis, widespread alopecia, dystrophic fingernails, and hypospadias. Initial ophthalmological evaluation revealed tiny bilateral Mittendorf dot lens opacities bilaterally that did not appear visually significant and no evidence of keratitis. In conjunction with his clinical findings, a failed newborn hearing screening and brain MRI demonstrating a posterior fossa cyst in continuity with the fourth ventricle, with absence of the inferior vermis and elevation of the torcula, prompted genetic testing. This revealed a de novo monoallelic heterozygous pathogenic variant (c.148G>A p.Asp50Asn) in the *GJB2* gene, confirming a diagnosis of KID syndrome. A thorough review of the patient's family history revealed no members with similar conditions.

Since his diagnosis, the patient's skin findings have been managed with a variety of topical therapies, including mupirocin 2% ointment, tretinoin, hydrocortisone, triamcinolone 0.025%, tacrolimus 0.03%, pimecrolimus 1%, fluocinolone acetonide 0.01% oil, and dilute bleach baths, along with systemic antihistamines such as hydroxyzine and cetirizine. At 2–1/2 years of age, he began to develop recurrent, painful nodulocystic lesions on the scalp, with purulent discharge from ear lesions and significant occipital lymphadenopathy. These lesions persisted despite repeated and extended courses of oral antibiotics.

He was subsequently started on acitretin (10 mg) three to four times per week, which resulted in a modest reduction in the frequency of scalp flares, though the lesions persisted, intermittently flared, and were accompanied by marked crusting, pain, and tenderness. In response to the family's preference to minimize acitretin use, 2% mefenamic acid cream was formulated and introduced, while acitretin was successfully reduced in frequency to 10 mg once per week. Mefenamic acid was compounded at a strength of 2% (2 g per 100 g of topical medication). The formulation of mefenamic acid provided to the patient was free of known skin irritants and sensitizers such as propylene glycol, but did include mild preservatives. A standard non‐comedogenic, hypo‐allergenic, non‐greasy, oil‐in‐water hydrophilic cream available to compounding pharmacies was used as the vehicle. A standard compounding scent selected by the family was added to mask the baseline odor of mefenamic acid. The patient was instructed to apply three fingertip‐lengths (~1.5 g) of cream, equivalent to 30 mg of mefenamic acid, twice a day to the scalp, face, and eyebrows.

**FIGURE 1:**
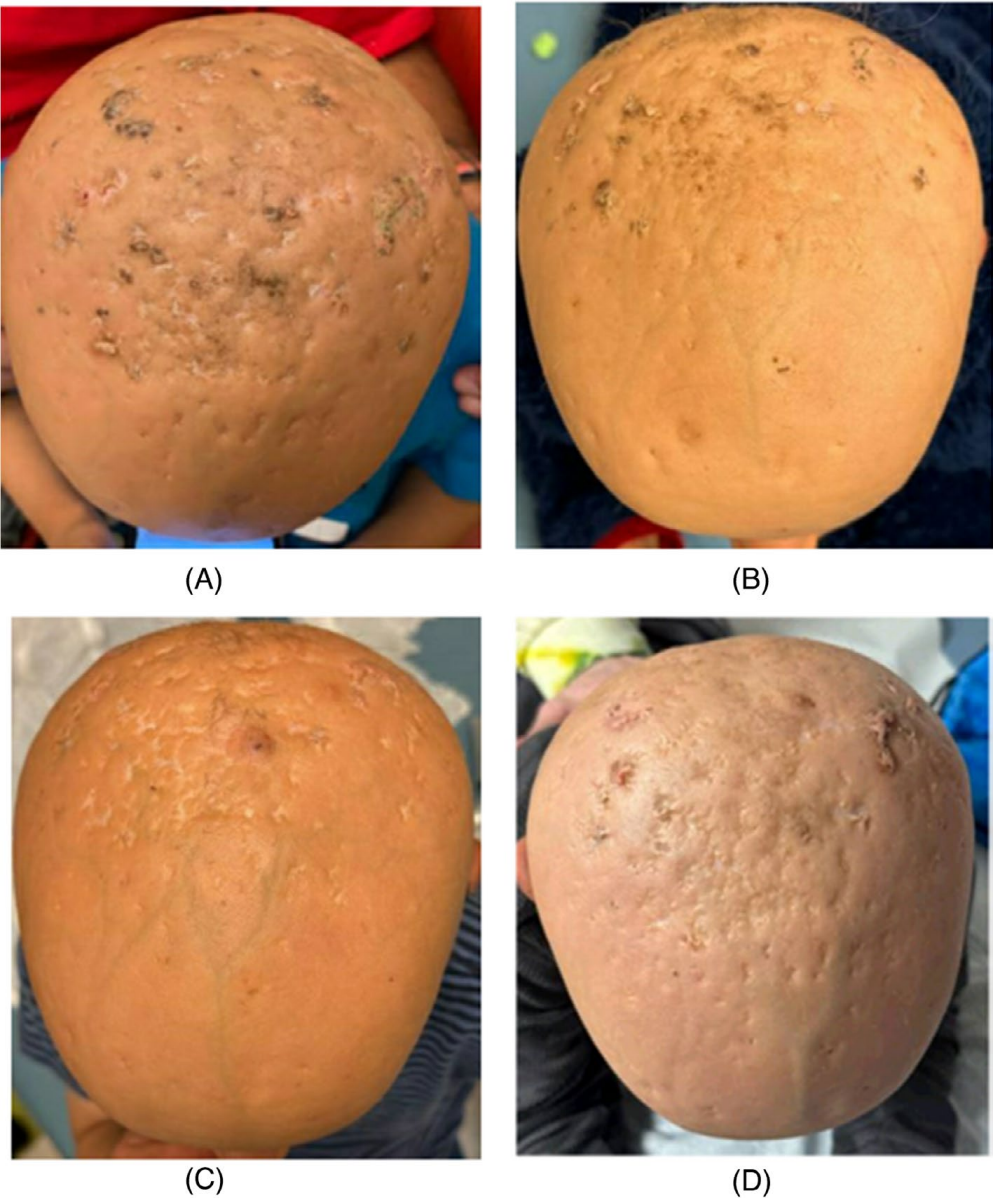
(A) Three months prior to starting mefenamic acid; ISS_scalp_ = 0.27. (B) One month prior to starting mefenamic acid; ISS_scalp_ = 0.045. (C) Nine months after initiating mefenamic acid; ISS_scalp_ = 0.009. (D) Fourteen months after initiating mefenamic acid; ISS_scalp_ = 0.009.

Three‐ and one‐month prior to initiating mefenamic acid, our patient was assigned Ichthyosis Scoring System scalp subscores (ISS_scalp_) of 0.27 and 0.045, respectively (Figure 1A,B). Following the application of mefenamic acid, there was noticeable improvement in all treated areas with decreased erythema and scaling. The patient has not required the use of fluocinolone or tretinoin since initiating mefenamic acid, and the family reported a significant reduction in the number of infectious nodules on the scalp that required mupirocin treatment. The patient has also seen an improvement in nodules on the eyebrows following the application of mefenamic acid to these areas. Nine months after initiating mefenamic acid, our patient was noted to have an improved ISS_scalp_ of 0.009 (Figure 1C). Screening laboratory markers—comprehensive metabolic panel (electrolytes, hepatic and renal function) along with lipid levels—were obtained as part of routine acitretin monitoring and showed no significant abnormalities associated with either low‐dose acitretin therapy or topical mefenamic acid treatment. To date, he continues to have improvement with topical mefenamic acid and follows closely with dermatology. Fourteen months after initiating mefenamic acid, our patient was noted to have a sustained improvement with an ISS_scalp_ of 0.009 (Figure 1D). During this time, acitretin has successfully been tapered from three times a week to two times a week and now to once a week following the initiation of the topical mefenamic acid. He also continues to follow with ophthalmology for bilateral keratitis that developed at 5 years of age, otolaryngology for hearing loss since infancy, and neurology for developmental monitoring in the setting of his congenital brain anomalies.

Though oral mefenamic acid is FDA approved in the United States, it has not been used in topical form for the treatment of skin disease. Our case presentation demonstrates the possible benefit of a novel treatment, mefenamic acid, in treating skin pathology associated with pediatric patients with KID syndrome.

## 3 Discussion

In this case report, we describe a 5‐year‐old boy with KID syndrome who exhibited significant improvement in his severe scalp lesions following treatment with topical mefenamic acid. Given the lack of effective treatments for KID syndrome and the risks associated with systemic therapies like acitretin, this innovative use of a compounded, topical formulation of an FDA‐approved nonsteroidal anti‐inflammatory drug raises the possibility of its use as a safe and effective therapeutic alternative for skin manifestations associated with KID syndrome, particularly in patients who may not respond adequately to systemic therapies such as isotretinoin or alitretinoin. Thus, clinicians treating patients with this condition should consider the possible advantages of compounded topical mefenamic acid as part of a topical treatment plan, at least for selected areas. Our patient administered cream that had the equivalent of ~30 mg of mefenamic acid applied topically twice daily. By comparison, oral mefenamic acid is typically administered to patients ≥ 14 years of age with a starting dose of 500 mg, followed by 250 mg every 6 h as needed. Nevertheless, we limited topical exposure to 10% body surface area since it was unclear whether systemic absorption in the context of an underlying genetic ichthyosis would be a concern. Further investigation may help us determine whether larger body surface areas can be tolerated safely.

The results from this case emphasize the importance of investigating alternative, safe, effective, and economically reasonable treatment options for rare diseases such as KID syndrome, for which standard therapy may be insufficient or associated with severe side effects. The purported mechanism of action of mefenamic acid is that it may inhibit aberrant connexin activity in keratinocytes and is consistent with the pathophysiology of KID syndrome, presenting a justification for further investigation.

Given that we report on a singular patient with KID syndrome, the conclusions from our study regarding the impact of mefenamic acid on epidermal pathology are limited. While our patient with KID syndrome has a variant in the *GJB2* gene, it is possible that patients with other variants may have different responses to mefenamic acid. It is warranted to validate these findings through treatment of additional patients in larger clinical trials. Longitudinal studies investigating the drug's safety and effectiveness in a diverse patient population are imperative to determine mefenamic acid's place in the treatment arsenal against KID syndrome and related dermatological conditions. If larger studies can validate the benefits of topical mefenamic acid, then it may be helpful to explore the potential role of systemic mefenamic acid in providing multisystem benefit, especially on the non‐epidermal, irreversible findings in KID syndrome such as hearing loss, though that would require more extensive and careful investigation given potential side effects, such as the possible impact of long‐term NSAIDs on renal function.

## Consent

Written informed consent was obtained from the patient's legal guardian for the publication of this case report and any accompanying images. The patient's identity has been kept anonymous to protect privacy, in accordance with applicable guidelines and regulations.

## Conflicts of Interest

Lars Brichta is an executive of Chemistry Rx, which created the compounded medication and provided it at no cost to the patient and family.

## Data Availability

Data sharing is not applicable to this article as no new data were created or analyzed in this study.
